# Effects of Voluntary Sodium Consumption during the Perinatal Period on Renal Mechanisms, Blood Pressure, and Vasopressin Responses after an Osmotic Challenge in Rats

**DOI:** 10.3390/nu15020254

**Published:** 2023-01-04

**Authors:** Cintia Y. Porcari, Agustina Macagno, André S. Mecawi, Agustín Anastasía, Ximena E. Caeiro, Andrea Godino

**Affiliations:** 1 Instituto de Investigación Médica Mercedes y Martín Ferreyra, INIMEC-CONICET-Universidad Nacional de Córdoba, Friuli 2434, Córdoba 5016, Argentina; 2 Laboratory of Molecular Neuroendocrinology, Department of Biophysics, Paulista Medical School, Federal University of São Paulo, São Paulo 04023-062, Brazil; 3 Instituto Universitario de Ciencias Biomédicas de Córdoba (IUCBC), Naciones Unidas 420, Córdoba 5016, Argentina; 4 Facultad de Psicología, Universidad Nacional de Córdoba, Boulevard de la Reforma & Enf. Gordillo Gómez, Córdoba 5000, Argentina

**Keywords:** perinatal programming, blood pressure regulation, vasopressin, TRPV1, voluntary sodium consumption

## Abstract

Cardiovascular control is vulnerable to forced high sodium consumption during the per-inatal period, inducing programming effects, with anatomical and molecular changes at the kidney, brain, and vascular levels that increase basal and induce blood pressure. However, the program- ming effects of the natriophilia proper of the perinatal period on blood pressure control have not yet been elucidated. In order to evaluate this, we studied the effect of a sodium overload challenge (SO) on blood pressure response and kidney and brain gene expression in adult offspring exposed to voluntary hypertonic sodium consumption during the perinatal period (PM-NaCl group). Male PM-NaCl rats showed a more sustained increase in blood pressure after SO than controls (PM-Ctrol). They also presented a reduced number of glomeruli, decreased expression of TRPV1, and increased expression of At1a in the kidney cortex. The relative expression of heteronuclear vaso- pressin (AVP hnRNA) and AVP in the supraoptic nucleus was unchanged after SO in PM-NaCl in contrast to the increase observed in PM-Ctrol. The data indicate that the availability of a rich source of sodium during the perinatal period induces a long-term effect modifying renal, cardiovascular, and neuroendocrine responses implicated in the control of hydroelectrolyte homeostasis.

## 1. Introduction

Sodium appetite is an innate behavioral mechanism for reaching an adequate physiological sodium level. However, consumption of sodium above or below the physiologi-cal limit has deleterious health consequences, and such consumption during pregnancy and/or the perinatal period has been shown to have long-term adverse effects on offspring [1,2,3,4,5,6,7,8,9,10,11,12,13,14,15].

In recent decades, the effect of a high/low sodium diet during the perinatal period on blood pressure and the health status of adult offspring has been widely investigated and demonstrated [2,3,4,5,6,7,8]. These studies have provided insights into how an early sodium-rich environment, by forced high sodium diet or hypertonic sodium solution as the only drink, induced evident long-lasting sodium/water intake imbalance and cardiovascular effects in the offspring [9,10,11,12,13]. This early experience with high sodium levels significantly in-creases the basal and induced blood pressure of adult offspring, due to undetermined programmed mechanisms [9,13,14]. For example, forced dietary high sodium intake dur-ing the perinatal period produced a reduction in the number of glomeruli, and an increase in components of oxidative stress and of the renin-angiotensin system. These elements may interact as inflammatory-oxidative responses, resulting in inadequate cardiovascular control [14,15].

Gestation is a natriophilic stage per se, in which hypertonic sodium solutions that are non-palatable in non-pregnant animals became appetitive for the dams during gestation, allowing them to achieve an increase in blood volume during pregnancy without altera-tion of hydroelectrolyte balance. However, it remains to be clarified whether this volun-tary consumption of hypertonic sodium solution during pregnancy and/or the perinatal period has any differential effects on cardiovascular regulation in adult progeny. We have previously demonstrated that voluntary sodium consumption during the perinatal period can modify homeostatic responses after challenges in water and sodium balance [16,17]. This early manipulation was able to: 1- affect sodium and water drinking response, increasing sodium preference; 2- reduce vasopressin synthesis in the SON; 3- decrease angiotensin type 1 receptor expression through the subfornical organ (SFO); and 4- modify the pattern of neuronal activity in such key structures as the nucleus of the solitary tract (NTS) and SFO. Thus, our previous data may suggest an altered osmotic regulation that affects sodium/water drinking balance and vasopressin response after hydroelectrolyte challenge.

The primary brain osmoreceptor regions are in the lamina terminalis (LT), a structure made up of the SFO and the organum vasculosum of the lamina terminalis (OVLT) cir-cumventricular organs (CVOs) [18,19,20]. Furthermore, the hypothalamic magnocellular neurons (MNC) are also intrinsically osmosensitive and lie in the supraoptic nucleus (SON) and paraventricular nucleus (PVN), which are responsible for releasing oxytocin (OT) and vasopressin (AVP) into the bloodstream [21,22,23]. Together these osmosensitive cells located in the central nervous system express the TRPV1 (transient receptor potential vanilloid type 1) channel, allowing them to respond to changes in osmolarity [24,25,26,27,28]. Our previous studies showed that the TRPV1 is a key channel in the modulation of sodium appetite and vasopressin response after body sodium depletion [27,28]. In recent years, the TRPV1 channel, at renal level, has been viewed as a protective buffer to the blood pressure response after a sodium overload (SO) challenge, as its activation decreases blood pressure and renal damage induced by SO in the DOCA-salt hypertension model, diminishing inflammatory responses [29,30,31]. It is thus possible that TRPV1 mechanisms are also affected by perinatal sodium programming, modulating, at least in part, the changes in osmoregulatory and cardiovascular responses to osmotic challenges. This work elucidates the effects of voluntary consumption of hypertonic NaCl during gestation and lactation on cardiovascular regulation in adult offspring, focusing on the renal and brain mechanisms involved.

## 2. Materials and Methods

### 2.1. Animals

**Figure 1:**
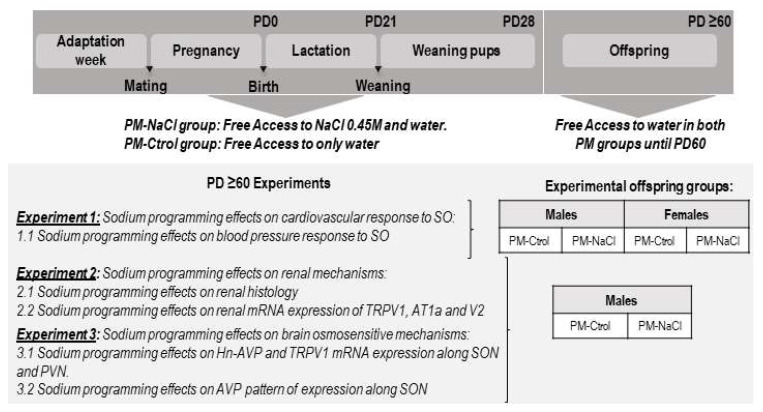
Schematic diagram showing the conditions of the dams and their offspring from adaptation week until the beginning of the studies. Note that from PD28 until PD60, male and female off-spring of PM-NaCl and PM-Ctrol groups were kept in standard conditions. From postnatal day 60 (PD ≥ 60), the offspring were subjected to different experimental protocols. In experiment 1, we analyzed the effects of sodium programming on cardiovascular response to SO, for which purpose the adult female and male offspring were anesthetized and submitted to a cannulation procedure to analyze the changes in MAP after SO. Given that only male rats showed differences in the MAP (see results section), only males were included in the analysis in the following experiments (focused on the possible underlying kidney and brain mechanisms of this effect). For experiments 2 and 3, a group of programmed and control animals at baseline and after SO challenge were decapitated, and their kidneys (experiment 2) and brains (experiment 3) were collected for gene expression determination. Another group of PM-NaCl and PM-Ctrol animals were perfused at baseline and after SO to perform kidney histological determination (Experiment 2) and to determine AVP immunofluorescence intensity along SON (experiment 3).

Female and male adult Wistar-derived rats, born and reared in the breeding colony of the Instituto Ferreyra (INIMEC-CONICET, UNC, Córdoba, Argentina) were used in these experiments. Females weighing 230–260 g and males 350–380 g, 60 days old and non- littermates, were individually housed in standard holding chambers (40 × 40 × 70 cm). Room lights were on for 12 h/day, beginning at 08:00 a.m., and temperature was controlled at 23 °C ± 1. All experimental protocols were approved by INIMEC’s appropriate animal care and use committee (Res #009/2019), following the guidelines of the international Public Health Service Guide for the Care and Use of Laboratory Animals (NIH Publications No. 8023, revised 1978). We complied with the ARRIVE guidelines. The protocol was executed according to Macchione et al., 2012, 2015 [16,17]. Briefly, 7 days before mating, female rats were randomly divided in two groups to receive each perinatal manipulation (PM): one group without manipulation (Female/Male PM-Ctrol group) with free access to distilled water and normal sodium diet (Purina Rat Chow, Argentina, containing approx. 0.18% NaCl) and the other group (Female/Male PM-NaCl group) that, in addition to free access to distilled water and normal sodium diet, had voluntary access to a hypertonic NaCl solution (0.45 M NaCl) (Figure 1). After one week of adaptation, one couple per cage was placed for mating in the same standard holding chamber until found sperm-positive, maintaining the hypertonic NaCl solution access in the PM-NaCl group. When pregnancy was confirmed (1–5 days), males were removed. Pregnant rats were maintained in the same holding chamber. Within 24 h after birth, litters were culled to ten pups, retaining both males and females in each litter. Litters with fewer than six pups were not included. Dams continued to receive their respective manipulation conditions until pups were weaned at postnatal day 21–22 (PD 21–22). After weaning, male pups were separated from female pups, and were maintained in the same conditions as their dam until reaching PD 28. Then, pups of both experimental conditions were kept in standard conditions of water and food until about 2 months of age (PD 60–65), when they were assigned to the corresponding experiments (see experimental protocols). In accordance with our previous report by Macchione et al., 2012 [17], maternal hypertonic sodium intake increased significantly during the pregnancy period compared to the adaptation week period (F(1, 22) = 36.902, *p* = 0.000001; PM-NaCl pregnancy 34.74 ± 3.11 mL/day vs. PM-NaCl adaptation 7.95 ± 2.6 mL/day). To avoid litter-specific effects, no more than three males or females per litter were used for the same condition. All the protocols were carried out between 8:00 a.m. and 2:00 p.m.

### 2.2. Sodium Overload (SO)

Male offspring rats (Males PM-Ctrol and Males PM-NaCl) aged 60 days old were subjected to SO. The animals were housed in cages without access to food and water for 40 min before SO. Lidocaine was administered subcutaneously (sc) to avoid pain and, one minute later, sc hypertonic sodium chloride solution (1 mL/100 b.w.; 2M NaCl) was administered. After 25–35 min of SO, both the basal animals (without overload) and the sodium overloaded animals were sacrificed for the different experiments.

### 2.3. Experimental Protocols

Experiment 1: Sodium programming effects on cardiovascular response to sodium overload (SO).

To assess adult offspring, males and females aged 60 to 65 days were submitted to the surgical cannulation procedure. The protocol was executed according to Caeiro and Vivas, 2008 [32]. Each animal was anesthetized with urethane (1.5 g/kg ip) and was im-planted with heparin–saline (50 U/mL)-filled polyethylene catheters (PE-50: 0.039 in. OD, 0.023 in. ID) in the right femoral artery. The arterial catheter was connected to a blood pressure transducer and Power Lab data-acquisition system (ADInstruments, Sydney, Australia).

A total period of 45 min was recorded: the first 10 min were the baseline record ing, and then we performed the SO challenge for 1 min, and then continued the recording for 40 min. We analyzed the changes with respect to baseline values of mean blood pressure (MAP; mm Hg), heart rate (HR; beats/min), diastolic and systolic pressure (DAP and SAP; mm Hg) (delta value). Given that the cardiovascular response was significantly different only in males, we continued analyzing renal and brain mechanisms only in this group in the following experiments (experiment 2 and 3).

Experiment 2: Sodium programming effects on renal mechanisms. To determine changes in kidney histology, a group of adult male offspring of PM-Ctrol and PM-NaCl groups (without any postnatal treatment) at 60–65 PND were anesthetized with thiopentone (100 mg/kg ip) and perfused transcardially with isotonic saline followed by 4% paraformaldehyde in 0.1 M phosphate buffer, pH 7.2. Kidneys were removed to analyze the anatomical and histological changes induced by the sodium perinatal programming. Kidneys were cut in coronal sections, which were stained with hematoxylin and eosin protocol to visualize the anatomical and histological changes in medulla and cortex size, glomeruli number and glomerular capillary/Bowman´s capsule ratio. Another group of PM-Ctrol and PM-NaCl groups were given SO, and 25 min later were decapitated, and kidney samples at cortex and medulla were taken to measure changes in Agtr1a, Trpv1, Avpr2 gene expression.

Experiment 3: Sodium programming effects on brain osmosensitive mechanisms. As detailed in Figure 1, experiment 3 determined sodium programming effects on key elements of central osmosensation at the hypothalamic level as, in our previous results, we found this involved hyperosmotic responses, with a reduction in total vasopressin expression after osmotic challenge along SON [16]. We therefore measured the expression of the osmosensitive channel TRPV1 and the heteronuclear AVP (Avp hnRNA: AVP synthesized de novo) along the PVN and SON from brains of the same groups of decapitated animals (PM-Ctrol and PM-NaCl) after SO of experiment 2. In order to confirm the data obtained from real time PCR in SON, another group of PM-Ctrol and PM-NaCl males (same animals of experiment 2) were anesthetized with thiopentone (100 mg/kg ip) and perfused transcardially with isotonic saline followed by 4% paraformaldehyde in 0.1 M phosphate buffer, pH 7.2. brains were removed and submitted to the immunofluorescence detection of AVP. The immunostained cells were visualized using a confocal microscope and scored intensity as described by Porcari et al., 2019 [33].

### 2.4. Relative mRNA Expression of Agtr1a, Trpv1, Avpr2, and hn-Avp in the Brain and Kidney

Immediately after decapitation, male offspring brains and kidneys were weighed and collected and frozen on dry ice in RNAse-free conditions and stored at −80 °C for Gapdh, Agtr1a, Trpv1, Avpr2 Mrna and Avp hnRNA determinations by Qpcr assay.

We performed transversal sections for cortex and medulla kidney (1500 μm) and cor-onal sections of 1200 μm for the supraoptic nucleus (SON, bregma: −0.96 mm to −1.44 mm) and the paraventricular nucleus (PVN, bregma: −1.44 mm to −1.92 mm). These were ob-tained from the frozen kidneys and brains through microtome cuts, and punches were performed using a stainless-steel needle (inner diameter 1.5 mm). The brain nuclei were identified and delimited according to a rat brain atlas [34].

RNA was isolated from the punched kidney and brain tissue using Trizol reagent (Invitrogen, Carlsbad, CA, USA), as directed by the manufacturer with some modifica-tions: RNA precipitation with isopropanol was performed overnight at –20 °C. Dnase-treated (Fermentas) RNA was quantified using a NanoDrop 2000 UV-Vis spectrophotom-eter and was then reverse-transcribed into Cdna (enzyme RTM-MLV –Promega). The gene expression to Gapdh, Agtr1a, Trpv1, Avpr2 in kidney, and Gapdh, Trpv1 and hn-Avp in brain was determined using Syber Green Real-Time PCR Master Mixes (Applied Biosystems™) in the Step One Real-Time equipment (StepOne™ Real-Time PCR System #4369074, Applied Biosystems, Foster, CA, USA). Primer sequences are in Table 1.

#### Calculations of Relative Gene Expression

The relative quantification was determined by the ΔΔCt method [35], where the fold change of mRNA content in the unknown sample relative to the control group was deter-mined by 2 − ΔΔCt (ΔΔCt = ΔCt unknown − ΔCt control). For each sample, the Ct was determined and normalized to the average of the housekeeping Gapdh. This gene is a constitutive and stable gene between groups, which allows its use as a control for this experiment. All samples were run in duplicate with the average CT used for each sample. The Ct of the calibrator group (the mean Ct of the naïve male adult rat) was then sub-tracted from each sample to give a Ct value. Relative quantification of the Agtr1a, Trpv1, Avpr2 and Avp hnRNA expression was normalized to the naïve male adult rat. Data are presented as mRNA relative to the control calibrator group.

### 2.5. Renal Histological Staining and Analysis Using Hematoxylin Eosin

A new group of PM-NaCl and PM-Ctrol basal adult animals were anesthetized with thiopentone (100 mg/kg ip) and perfused transcardially with isotonic saline followed by 4% paraformaldehyde in 0.1 M phosphate buffer, pH 7.2. Kidneys were removed, kept in paraformaldehyde solution overnight, and stored at 4 °C in 30% sucrose until processed.

Kidneys were cut into 40 µm coronal sections using a microtome and mounted on gelatinized slides. All sections were stained with hematoxylin (Mayer’s Hematoxylin, Bi-opack, cod. 2000948900) and eosin (Eosin yellowish 0.5%, Biopack, cod. 2000110500) and mounted with DPX slide mounting medium and coverslips. Histological images were dig-itized and saved at 5×, 10×, and 20× microscope magnifications, using a Zeiss microscope equipped with a Leica DC 200 digital camera connected to a computer.

The analysis of each parameter was performed blind with the NIH Image J software (National Institutes of Health, Bethesda, MD,66, Fiji (RRID:SCR_002285); https://fiji.sc/-accessed on 10 November 2022). The images with 5× magnification were used to measure the size of the renal cortex, and the 10× sections to count the total glomeruli and glomeruli per mm^2^ of the image. In the 20× images, we analyzed the size of the glomerular capillaries and of the Bowman capsule and calculated the relationship between both. In this experiment, we worked with kidneys from PM-NaCl and PM-Ctrol basal animals (n = 6), of which at least three sessions (images) of each were quantified, to ensure that the average number was as precise as possible.

### 2.6. Immunofluorescence

Rats were anesthetized with thiopentone (100 mg/kg ip) and perfused transcardially with ~100 mL of 0.9% saline solution followed by ~400 mL of 4% paraformaldehyde in 0.1 M phosphate buffer (PB, pH 7.2). The brains were removed, fixed in the same paraform-aldehyde solution overnight and then stored at 4 °C in PB containing 30% sucrose. Serial coronal sections (20 µm) of supraoptic nucleus were made in a microtome cryostat. Free-floating sections were washed with 0.01 M PB (pH 7.2) and permeabilized with 0.2% Tri-ton. Nonspecific staining was blocked with 10% normal horse serum (NHS) for 1 h. Next, the tissues were incubated with mouse anti-AVP- Neurophysin primary monoclonal an-tibody (PS41 1:200) generously donated by Dr. Harold Gainer (NIH, Bethesda, MD, USA) [36] at 4 °C for 24 h in 0.1 M PB containing 2% NHS. The sections, protected from light, were then incubated with the secondary antibody (1:500) Alexa 488-labeled anti-mouse for 2 h at room temperature in 0.1 M PB containing 2% NHS. After incubation, the sections were washed with 0.01 M PB followed by washing with 4 M calcium carbonate. Then, the sections were mounted onto chromo-alum-coated slides, air dried, and cover slipped with Fluoromount (Sigma-Aldrich, St Louis, MO, USA). The immunostaining specificity was characterized by preincubating the antiserum with 30 or 50 µg of the immunogenic peptide and staining the brain tissue as described above.

The immunostained cells were visualized using a confocal Zeiss LSM 800. Quantita-tive analysis of immunofluorescence images captured by ImageJ software [37,38]. The average of immunofluorescence of five cells within the area of interest in each section was calculated maintaining the gain and the power of the laser constant in arbitrary units (a.u.).

### 2.7. Statistical Analysis

Results were expressed as mean (M) ± standard error (S.E.) and a level of *p* < 0.05 was considered statistically significant. The normality of the data was assessed with the Shapiro–Wilk test. Blood pressure and heart rate data were analyzed by using one-way ANOVA with repeated measurements: perinatal manipulation (PM-NaCl and PM-Ctrol) and time post-NaCl-injection (during 40 min). Real-time PCR and immunofluorescence results were analyzed by two-way analyses of variance (ANOVA): sodium condition (Basal and SO) and perinatal manipulation (PM-NaCl and PM-Ctrol) as main factors in randomized blocks. Renal histology results were analyzed with an unpaired Student’s t test. Analyses were performed using STATISTICA 8 software. Statistically significant interactions were further analyzed using the Tukey test (type I error probability was set at 0.05). The partial eta-squared (η2p) was used to describe effect sizes of the ANOVAs, and was interpreted using the following guidelines (small (η2p  =  0.01–0.05), medium (η2p  =  0.06–0.13), and large (η2p  ≥  0.14) [39]).

## 3. Results

### 3.1. Sodium Programming Effects on Cardiovascular Response to SO

**Figure 2:**
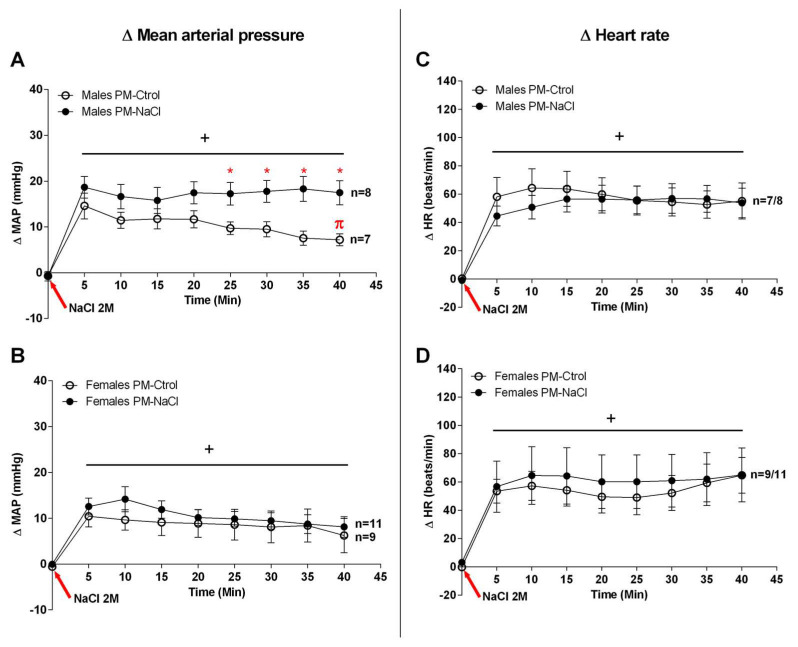
Sodium programming effect on mean blood pressure (MAP) and heart rate (HR) in response to sodium overload (SO). Line graphs show the effect of SO on mean arterial pressure changes (MAP, (**A**,**B**) panel) and heart rate (HR, (**C**,**D**) panel) in male ((**A**,**C**) panel) and female rats ((**B**,**D**) panel) of both PM conditions (PM-Ctrol and PM-NaCl). Data are plotted as the changes (Δ) in MAP and HR compared with baseline values obtained 10 min before NaCl 2M injection. Number of animals in each group: Males: PM-Ctrol (n = 7) and PM-NaCl (n = 8); Females PM-Ctrol (n = 9) and PM-NaCl (n = 11). * *p* < 0.05 compared to PM-Ctrol group at 25, 30, 35 and 40 min after NaCl 2M injection. + *p* < 0.05 vs. baseline at time zero. π *p* < 0.05 compared to 5 min of PM-Ctrol (higher point to baseline).

The statistical analysis indicated that both male and female rats of the PM-Ctrol and PM-NaCl groups showed an increase in blood pressure in response to SO (males F8, 136 = 19.557, *p* < 0.001 and females F8, 136 = 19.557, *p* < 0.001; NaCl-injection factor). However, male offspring of the PM-NaCl group showed higher MAP levels than the PM-Ctrol, from 25 min post infusion (interaction: F8, 104 = 3.685, *p* = 0.001) (Figure 2A); this pattern was also observed in DAP (F8, 120 = 2.173, *p* = 0.034), but not in SAP (F8, 104 = 1.591, *p* = 0.136). The PM-Ctrol males also started to recover MAP at 35–40 min after SO, but this response was absent in PM-NaCl rats. That is, our results demonstrated a similar increase in MAP immediately after the challenge; however, the recovery response is altered in sodium-programmed males. On the other hand, although an increase in mean arterial pressure was observed in females (F 7.98 = 12.971; *p* < 0.001) (Figure 2B), no differences were found among PM-NaCl and PM-Ctrol groups. PM-Ctrol and PM-NaCl females also did not show any differences in SAP (F8, 136 = 0.454, *p* = 0.886) or DAP (F8, 200 = 0.857, *p* = 0.554).

An equal increase in the HR was observed in both groups (PM-Ctrol and PM-NaCl) of males (F 7.140 = 28.348; *p* < 0.001) and females (F 7.112 = 19,340; *p* < 0.001), with no perinatal programming effect (Figure 2C,D).

### 3.2. Sodium Programming Effects on Renal Mechanisms

#### 3.2.1. Sodium Programming Effects on Renal Histology

**Figure 3:**
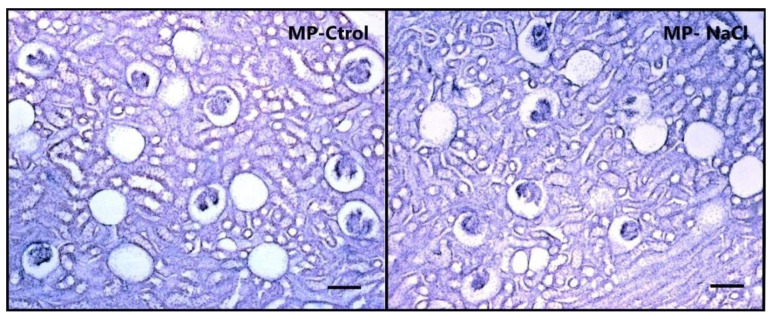
Sodium programming effects on kidney histology. Photomicrographs showing the histo-logical pattern of glomeruli in the renal cortex stained with hematoxylin and eosin from basal PM Ctrol (**left**) and PM-NaCl (**right**) animals. Photographs are shown at 5X magnification. Scale bar: 100 μM.

Renal histological analysis revealed a significant effect of the number of total glomer- uli (5× microscope magnifications), and per mm^2^ (t-value glo total = 3.07; *p* = 0.012. t-value glo × mm^2^ = 3.21; *p* = 0.007), showing that animals with maternal programming (PM-NaCl) have a lower number of renal glomeruli than controls (PM-Ctrol) (Table 2, Figure 3).

#### 3.2.2. Sodium Programming Effects on Renal mRNA Expression of TRPV1, AT1a and V2 Receptor

**Figure 4:**
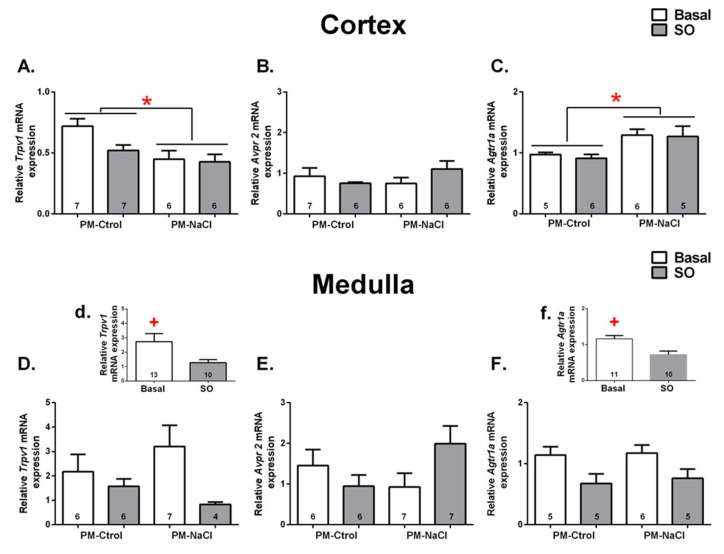
Sodium programming effects on renal mRNA expression of receptor potential vanilloid type 1 (Trpv1), vasopressin receptor type 2 (Avpr2), and angiotensin receptor type 1a (At1a), in response to sodium overload (SO). Relative levels of Trpv1 (**A**,**D,d**), Avpr2 (**B**,**E**), and Agtr1a (**C**,**F**,**f**) mRNA in renal cortex (**A**–**C**) and medulla (**D**,**d**,**E**,**F**,**f**) of male PM-Ctrol and PM-NaCl animals under basal conditions (basal), and after SO. The numbers inside the bars represent the number of animals in the group. + significant difference at *p* < 0.05 between SO and basal groups; * significant difference at *p* < 0.05 between PM-Ctrol and PM-NaCl groups.

Trpv1: Trpv1 channel mRNA expression was analyzed in the renal cortex and me-dulla under basal conditions and after SO in PM-Ctrol and PM-NaCl animals. In cortex, a significant effect of perinatal manipulation can be observed (F 1.22 = 8.74, *p* = 0.009, η2p = 0.29, Figure 4A), but no effect of interaction and/or sodium condition. Regardless of sodium status, PM-NaCl animals showed lower mRNA expression of the Trpv1 channel than the PM-Ctrol group.

In the renal medulla, there was a significant decrease in Trpv1 channel mRNA expression after SO, regardless of perinatal treatment. Here, we observed a statistical differ-ence in the sodium condition factor (F 1.19 = 4.68, *p* = 0.044, η2p = 0. 20, Figure 4D,d), but no effect of the perinatal manipulation factor and/or interaction.

Avpr2: No significant differences were found in expression of Avpr2 mRNA in the renal medulla and cortex (Figure 4B,E).

Agtr1a: Agtr1a mRNA expression was analyzed in the renal cortex and medulla under basal conditions and after SO in PM-Ctrol and PM-NaCl animals. As can be seen in Figure 4C in the renal cortex, regardless of the sodium condition, there was a significant effect of the perinatal manipulation factor (F 1.18 = 9.20, *p* = 0.009, η2p = 0.39). Agtr1a mRNA expression was increased in the PM-NaCl group compared to the PM-Ctrol group (Figure 4C).

In renal medulla, there were statistical differences in the sodium condition factor (F 1.17 = 9.64, *p* = 0.009, η2p = 0.009, Figure 4F), but without any effect of the perinatal manipulation factor and/or interaction. Figure 4F,f shows a significant decrease in Agtr1a mRNA expression after SO, regardless of perinatal treatment.

### 3.3. Sodium Programming Effects on Brain Osmosensitive Mechanisms

#### 3.3.1. Hn-Avp and Trpv1 mRNA Expression along SON and PVN

**Figure 5:**
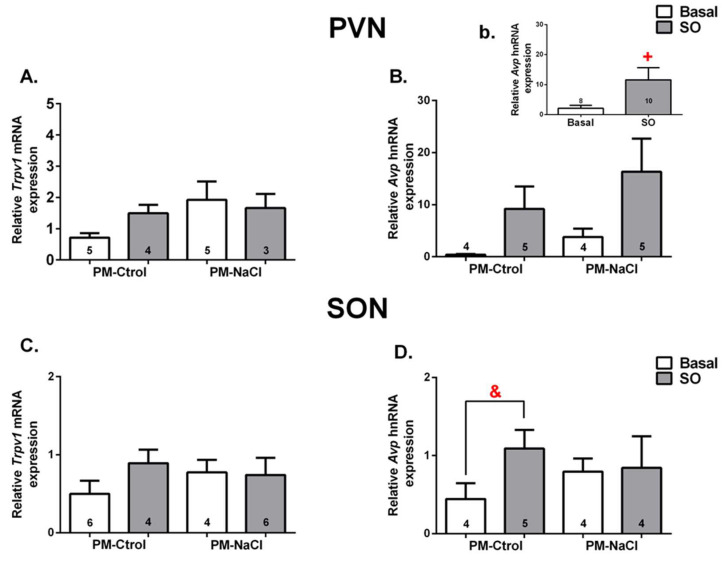
Sodium programming effects on brain expression of receptor potential vanilloid type 1 (Trpv1, mRNA) and heteronuclear vasopressin (Avp hnRNA) in response to sodium overload (SO). Relative levels of Trpv1 (**A**,**C**) and Avp hnRNA (**B**,**b**,**D**) in the paraventricular nucleus (PVN; (**A**,**B**)) and supraoptic nucleus (SON; (**C**,**D**)) of male PM-Ctrol and PM-NaCl animals under basal conditions (basal) and after SO. Values are mean ± SE. The numbers inside the bars represent the number of animals in the group. The numbers inside the bars represent the number of animals in the group. + significant difference at *p* < 0.05 between SO and basal groups. & significant difference at *p* < 0.05 between PM-Ctrol basal and PM-Ctrol SO groups.

The effect of perinatal manipulation on Avp hnRNA and Trpv1 mRNA expression was analyzed under basal conditions and after sodium overload (SO) in the supraoptic and paraventricular nuclei. In PVN, no significant differences were found in the mRNA expression of Trpv1 (Figure 5A). However, a significant effect of sodium condition factor on the expression of Avp hnRNA was observed in this nucleus (sodium condition (Basal vs. SO) F 1.14 = 6.57; *p* = 0.026; η2p = 0.29; Figure 5B) regardless of the perinatal manipulation effect. As expected, hyperosmolarity induced a significant increase in Avp hnRNA expression compared to the basal group. It is important to note that the perinatal manipulation factor and/or interaction between both factors produced no significant effects in this case.

Throughout the SON, we found a significant effect of the interaction between sodium condition and perinatal manipulation factors on RNA expression of Avp (interaction: F 1.13 = 7.35; *p* = 0.022; η2p = 0.055) (Figure 5D). PM-Ctrol animals with SO significantly increased hnRNA expression of Avp in relation to basal PM-Ctrol animals. However, we did not observe any differences in Avp hnRNA expression between PM-NaCl groups (basal vs. SO), or significant effects for the expression of the Trpv1 channel in the SON (Figure 5C).

#### 3.3.2. Sodium Programming Effects on AVP Pattern of Expression along SON

**Figure 6:**
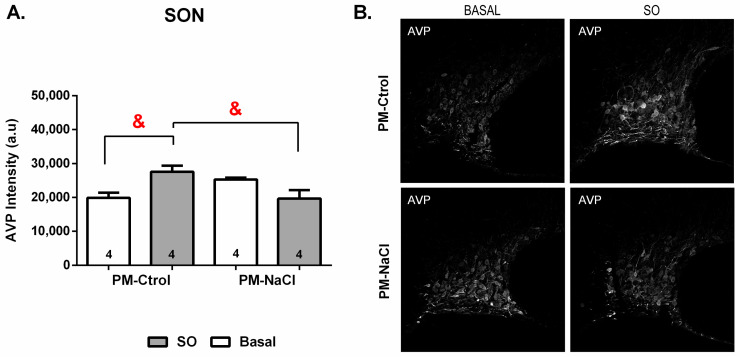
(**A**)-Sodium programming effects on immunofluorescence for AVP in the hypothalamic supraoptic nucleus (SON). The numbers inside the bars represent the number of animals in the group. Values are means ± SE. & significant difference at *p* < 0.05 between PM-Ctrol SO vs. PM-Ctrol Basal and PM-NaCl SO. (**B**)-Photomicrography showing the immunofluorescence pattern of AVP-positive cells within the SON.

The immunofluorescence analysis revealed significant effect of the interaction between sodium condition and perinatal manipulation factors on of AVP positive cells of PM-Ctrol and PM-NaCl animals in the SON (at 20×) at baseline or after SO (F 1.12 = 10.093, *p* = 0.008). As can be seen in Figure 6, PM-Ctrol animals show an increase in AVP fluorescence intensity after SO compared to PM-Ctrol Basal and PM-NaCl SO animals (Figure 6).

## 4. Discussion

Our results provide new information on the programming effects of voluntary hy-pertonic sodium consumption during the perinatal period on the homeostatic capacity of adult offspring. The data show that increasing natriophilia during gestation and early in-fancy changes blood pressure regulation under an osmotic challenge, affecting renal and brain mechanisms.

Blood pressure increases in response to high body sodium levels [40], and we ob-served that the SO heightened the MAP in both PM-NaCl and PM-Ctrol animals. NaCl-programmed males showed a sustained increase in blood pressure compared to PM-Ctrol male rats, while no differences were observed between female program groups in the in-creased MAP profile induced by SO. Considering that estrogen and sexual chromosome factor have a protective effect against hypertension in females [41,42,43], their cardiovas-cular response may have an important hormonal and chromosomal influence that masks the programming effect in females at reproduction.

Intrauterine adversity or prenatal or postnatal perturbation in preterm infants may result in a reduced nephron number. Any insult occurring prior to the completion of nephrogenesis likely compromises renal growth and produces a long-lasting effect on final renal potential function. The forced consumption of a high-salt diet during the perinatal period, when nephrogenesis occurs, reduces the number of nephrons in the offspring [14,44]. This insult may also be associated with an increase in the glomerulosclerosis index [45] and these early effects also induce a long-term blood pressure increase in the offspring [14]. In this context, we observed that voluntary sodium consumption during this period also reduced the number of glomeruli. The severity of alterations during fetal development depends on the nature, timing, duration, and severity of the renal insult. Thus, the decrease observed in our model, where sodium was a free choice with water available, showed a slighter decrease in nephron numbers than that observed under forced high-salt intake [14,15,44]. Environmental cues also influence a broad genetic program that results in renal perinatal programming, affecting the expression of several genes.

It has been postulated that essential hypertension must involve a renal expression of factors favoring sodium retention, thereby preventing pressure-induced natriuresis from restoring blood pressure toward normal levels [46]. The RAS is a key modulator of renal function and nephrogenesis [47]; therefore, changes in RAS during fetal and early postnatal life may have long-term repercussions on renal performance [45,47]. Studies, both by immunohistochemistry and by expression of mRNA, have demonstrated localization of AT1R in the glomerular afferent arteriole (blood vessels), at the level of the proximal and collecting tubules of the external medulla [48]. Similarly, we observed an increased expression of At1a receptor along the kidney cortex in sodium-programmed animals. In the same kidney region, we also found a decrease in TRPV1 expression in sodium-programmed adult offspring. In contrast to the role of At1, the TRPV1 channel has been shown to play an important role in preventing the development of salt- sensitive hypertension [49], by increasing the glomerular filtration rate and decreasing inflammatory responses [29,30,31,50,51]. Taken together, the reduction in renal Trpv1 and increase in At1a expression induced in programmed animals may decrease the efficacy of excreting sodium and water and may modulate the inflammatory response (infiltration of macrophages and lymphocytes, and release of proinflammatory cytokines as tumoral necrosis factor and interleukin 6) and/or oxidative stress, respectively, making these animals more susceptible to maintaining increased blood pressure. On the other hand, these genes’ mRNA expression at renal medulla level was not influenced by the perinatal manipulation, and thus we observed decreased expression of At1a receptors and Trpv1 channels after acute SO in adults.

Peripheral SO information reaches the brain through the nucleus of the solitary tract (NTS) (where information regarding high/low pressure receptors arrives) and CVOs of the lamina terminalis (SFO and OVLT) (where osmotic and sodium changes are detected). This information is integrated and projected to other areas to modulate blood pressure, AVP release, autonomic system and drinking behavior, to reestablish homeostasis. It has been demonstrated that this network also recruits the hypothalamic paraventricular and median preoptic nuclei to control the sympathetic reflex through the presynaptic rostral ventrolateral medulla (RVLM) and the preganglionic spinal medulla [52,53,54,55,56]. The different brain areas and pathways of this circuit may be more or less vulnerable to the perinatal programming effect of sodium, resulting in intrinsic changes at the different cellular, molecular, and system (i.e., vasopressinergic, angiotensinergic) levels, altering normal function and/or the mechanisms involved in achieving fluid and electrolyte homeostasis.

Our present results indicate a similar magnitude in changes in MAP after the challenge, but the recovery response is altered or delayed in sodium programmed males. Our previous results demonstrated that hypertonic early perinatal exposure to sodium decreased neuronal activity in the NTS and SFO areas (shown by Fos-immunoreactivity) induced by SO stimulus [16]. These areas are involved in blood pressure and osmolarity control respectively. The decreased activity in PM-NaCl animals may produce less stimulation of caudal ventrolateral medulla (CVLM) and this may result in a less inhibition of RVLM, inducing an increased sympathetic activity in PM-NaCl rats compared to PM-Ctrol [52,53]. Similarly, electrophysiological evidence has shown that there are “hypertonic neurons” that also receive baroceptor information which activates the blood pressure control circuit [57], and lesion of the NTS induces a greater increase in blood pressure after SO than in sham-NTS animals [58]. Taken together, these data suggest that alterations of areas involved in pressure regulation and in the perception of osmolarity and/or baroreception, such as SFO and NTS, in the face of cardiovascular challenges, may affect MAP regulation. These imbalances may affect sympathetic control (lowering the activity of the brainstem sympathetic circuit NTS and SFO), modulating the changes in pressure observed after an osmotic stimulus in programmed rats.

Our present and previous findings show that the vasopressinergic system along the SON is also affected by perinatal sodium exposure, slowing down the increase in the ex-pression of AVP neuropeptide and total and de novo synthesized (heteronuclear) RNA expression of Avp after the infusion of hypertonic NaCl solution [16]. The secondary effect of this AVP-refractory response may translate to less ability to recover renal water to reestablish osmolarity. Thus, correction of hyperosmolarity could be delayed, stimulating a blood pressure increase for longer, as observed in sodium-programmed animals.

## 5. Conclusions

In summary, our results demonstrate that voluntary hypertonic sodium consump-tion during the perinatal period affects the cardiovascular homeostasis that occurs after pressor stimulation. Its effects could be due to the brain AVP/sympathetic circuit and/or renal factors. This data is interesting because maternal sodium consumption influences blood pressure in the next generation, the pregnancy stage itself induces increased natriophilia, and sodium consumption is a modifiable component in the mother’s and/or newborn’s diet that affects the offspring’s health in adult life. These experimental animal models add valuable information to aid doctors’ recommendations to patients about the control of salt intake levels and hydroelectrolyte balance.

## Figures and Tables

**Table 1:** Primer pairs for Agtr1a, Avpr2, Trpv1, Avp hnRNA, and Gapdh mRNA.

Gene	Forward Primer 5′-3′	Reverse Primer 5′-3′	Product Size (bp)
Gapdh	TGTGAACGGATTTGGCCGTA	ATGAAGGGGTCGTTGATGGC	20
Agtr1a	AACCCTCTGTTCTACGGC	ACCTGTCACTCCACCTCA	18
Trpv1	TTCACCGAATGGGCCTATGG	TGAC- GGTTAGGGGTCTCACT	20
Avp hnRNA	GACGCAA- GAGGGCCACATC	CTCTCCTAGCCCATGA CCCTT	20/19
Avpr2	AAGCTCCTCTGGAAAGACCC	CAAAGCAGGCTACGCAACTC	20

**Table 2:** Size and basal histological values of PM-Ctrol and PM-NaCl kidneys.

Factors Analyzed	PM-Ctrol Mean ± SE	PM-NaCl Mean ± SE	*p*-Value
Kidney size (g/100 gpc)	0.43 ± 0.01 (n = 23)	0.42 ± 0.01 (n = 26)	0.204
Renal cortex size (mm)	14.73 ± 0.79 (n = 6)	15.02 ± 0.42 (n = 7)	0.745
N° Total of glomeruli (5× microscope magnifications)	14.54 ± 0.42 (n = 6)	12.39 ± 0.56 (n = 6)	0.012 *
N° of glomeruli/mm^2^	3.0 × 10^−2^ ± 9.1 × 10^−4^ (n = 6)	2.0 × 10^−2^ ± 9.8 × 10^−4^ (n = 6)	0.007 **
Glomerular capillary/Bowman’s capsule (%)	83 ± 4 (n = 7)	80 ± 2 (n = 7)	0.559

Values are means ± SE, * *p* < 0.05 and ** *p* < 0.01 significantly different PM-NaCl from PM-Ctrol group.

## Data Availability

The data presented in this study are available on request from the authors.
